# Real-World Comparison of Telemonitoring Versus Conventional Care in Patients With Chronic Obstructive Pulmonary Disease and Those With Asthma—Impact on Clinical Outcomes and Patient Characteristics: Retrospective Cohort Study

**DOI:** 10.2196/66743

**Published:** 2025-08-14

**Authors:** Lidy Aaltje Catharina Roubos, Heleen Westland, Niesje Lieset Hulstein-Brink, Rosalie Constance Visser, Jan Willem K van den Berg, Jobbe PL Leenen

**Affiliations:** 1 Connected Care Centre, Isala, Dr van Heesweg 2Zwolle, 8025 AB, The Netherlands, 31 0886245000; 2 Nursing Sciences, Program in Clinical Health Sciences, Utrecht University, Utrecht, The Netherlands; 3 Department of Orthopaedic Surgery, OLVG, Amsterdam, The Netherlands; 4 Department of General Practice & Nursing Science, Julius Center, University Medical Center Utrecht, Utrecht, The Netherlands; 5 Department of Pulmonary Medicine, Isala, Zwolle, The Netherlands; 6 Research Group IT Innovations in Healthcare, Windesheim University of Applied Sciences, Zwolle, The Netherlands

**Keywords:** chronic obstructive pulmonary disease, pulmonary disease, chronic obstructive, asthma, pulmonary disease, telemedicine, telemonitoring, real-world evidence, mHealth

## Abstract

**Background:**

Chronic obstructive pulmonary disease (COPD) and asthma belong to the most common chronic diseases and their incidence continues to rise. Many patients experience exacerbations leading to hospitalization, impacting quality of life and straining health care systems. Telemonitoring emerged as a substitute for outpatient clinic visits, aiming to intervene early and prevent hospitalization. However, studies evaluating telemonitoring are conducted in controlled settings and may not fully reflect real-world conditions. Real-world evidence is needed to understand how telemonitoring functions in routine clinical practice.

**Objective:**

This study aims to describe and compare patient characteristics and clinical outcomes of patients with COPD or asthma who received telemonitoring versus conventional care based on real-world data.

**Methods:**

An observational cohort study with retrospective data collection was conducted with data from newly diagnosed patients with COPD or asthma who received telemonitoring or conventional care with up to 1-year follow-up. Outcomes included patient characteristics, COPD- or asthma-related hospitalizations, emergency department visits, exacerbations, and outpatient clinic visits. The telemonitoring intervention involves a mobile app where patients weekly complete the Asthma Control Questionnaire or the Clinical COPD Questionnaire, allowing nurses to intervene if scores indicate deterioration. The app serves as a substitute for outpatient clinic visits for patients with COPD, while patients with asthma use it as a complement to these visits.

**Results:**

The study included 614 patients in conventional care and 96 patients in telemonitoring. Telemonitoring users are younger, predominantly female, rarely current smokers, and have fewer comorbidities. More patients with asthma used telemonitoring than patients with COPD. Patients using telemonitoring showed more moderate exacerbations (incidence rate ratio [IRR] 2.15, 95% CI 1.16‐3.98; *P*=.02). Although telemonitoring users experienced fewer hospitalizations, this was not significant after adjusting for confounders (IRR 0.68, 95% CI 0.15‐3.11; *P*=.62). Telemonitoring users had more telephone and screen-to-screen consultations (IRR 7.16, 95% CI 5.47‐9.36; *P*<.001), but outpatient clinic visits remained consistent across both groups (IRR 1.19, 95% CI 0.88‐1.62; *P*=.27).

**Conclusions:**

Patient characteristic differences and clinical outcome differences were identified between telemonitoring and conventional care. Although telemonitoring facilitated earlier initiation of treatment, it did not lead to fewer hospital or outpatient clinic visits. More insight is needed into factors influencing participation in telemonitoring to better serve current users and improve accessibility for nonusers. Patients should be provided with additional guidance on effectively using the communication channels offered by telemonitoring. This may encourage them to use these methods instead of attending outpatient clinic visits. Additionally, when implementing telemonitoring, it is essential to critically evaluate and redesign care processes to prevent unnecessary health care use.

## Introduction

Chronic obstructive pulmonary disease (COPD) and asthma belong to the most common chronic diseases and their incidence continues to rise [1]. The World Health Organization states that COPD is the third leading cause of death globally, resulting in 3.23 million deaths in 2019 [2]. Asthma is a prevalent disease, causing more than 455,000 deaths in 2019 [3].

Both COPD and asthma are diseases that progress variably. While some patients have extended periods without symptoms, others face exacerbations or acute worsening periods [4]. These exacerbations may lead to repetitive hospitalizations that affect the quality of life, prognosis, and the financial burden on health care systems [5-8].

COPD and asthma are more prevalent among the aging population [9]. As people are living longer and the shortage of nurses and physicians continues to grow, we are facing a forthcoming challenge in meeting the rising demand for health care services for patients with these diseases [10-12]. In response to the challenges of this growing demand, health care professionals and policy makers are compelled to explore innovative and cost-effective interventions, such as telemonitoring.

Telemonitoring is a telemedicine strategy that emerged in the early 1990s [13]. It enables patients to self-manage their health from the comfort of their homes, which can be used as an alternative to outpatient clinic visits or complement them. Telemonitoring involves the utilization of telecommunication technology to transmit health-related data from patients’ homes to health care facilities [14]. Through continuously monitoring the health of patients with COPD or asthma, for example, with questionnaires or vital signs, telemonitoring serves the purpose of alerting when clinical changes occur with potential risk for the patient and thereby timely assessing disease decompensation [14]. This facilitates early interventions, aiming to reduce hospitalizations and mortality rates [15,16]. Additionally, telemonitoring aims to support patient education and provide patients greater insight into their own health, enhancing disease control and quality of life [17,18].

Numerous randomized controlled trials (RCTs) and systematic reviews have investigated clinical outcomes of telemonitoring for patients with COPD or asthma. Some systematic reviews did not find evidence of reduced hospitalizations, emergency department (ED) visits or exacerbations, or conflicting evidence [16,19-21]. Other systematic reviews showed promising results, including a decrease in COPD- or asthma-related hospitalizations [16,22,23] and a reduced frequency of COPD- or asthma-related ED visits [22,24]. Despite not all findings being consistently promising, the absence of reported negative effects suggests that telemonitoring may be considered a safe alternative to conventional care [16,20].

Real-world evidence (RWE) RCTs evaluate telemonitoring in a tightly controlled setting, using strict inclusion and exclusion criteria. These conditions may lead to findings that do not fully represent the diverse populations and scenarios that are seen in routine daily practice. In clinical practice, patients make choices involving telemonitoring based on personal preferences, clinical factors, and other individual circumstances [25]. Consequently, outcomes in routine daily practice may differ from those observed in RCTs [26]. To overcome this gap between clinical research and clinical practice, RWE studies are upcoming. RWE is evidence based on medical data generated during routine patient care [26]. RWE studies use these real-world data to assess the effectiveness of an intervention when applied in the practical context of clinical care, providing a deeper understanding of how the intervention functions in this setting in which patients ultimately use it [27-29]. With telemonitoring apps being widely implemented in many hospitals, this presents a timely opportunity to evaluate their use and effectiveness in routine clinical care. To complement the existing evidence from RCTs, this study aims to describe and compare patient characteristics and clinical outcomes of patients with COPD or asthma who received telemonitoring versus conventional care based on real-world data.

## Methods

### Study Design

An observational cohort study with retrospective data collection was conducted at a large regional hospital with 900 beds in the Netherlands. To enhance reporting quality, the STROBE (Strengthening the Reporting of Observational Studies in Epidemiology) guideline for cohorts was used [30].

### Participants

Patients were eligible for inclusion if they were newly diagnosed with COPD or asthma and received either telemonitoring or conventional care for a minimum of 6 months of follow-up at the outpatient clinic between January 1, 2023, and March 31, 2024. Data collection started 6 weeks after the diagnosis of COPD or asthma, as this was the point on which telemonitoring was introduced. From this moment (baseline), patients were followed until the end of the study period (March 31, 2024) or until they were referred back to primary care. For patients with COPD, a maximum follow-up time of 1 year was maintained, aligning with the duration of the COPD care pathway before patients are referred back to primary care. For patients with asthma, the maximum follow-up time was 8 months, consistent with their care pathway. Extending follow-up beyond these periods could result in missing data due to patients returning to their general practitioner in case of symptom worsening. In addition, the selected durations account for seasonality, which can significantly influence the course of both conditions. Although patients with asthma could not be followed for a full year, they were enrolled throughout different times of the year, ensuring that seasonal variation was adequately represented in the dataset.

### Intervention

The telemonitoring intervention used in this study involves a mobile app, designed to improve patients’ knowledge of their disease and promote self-management. Conventional care for patients with both COPD and asthma exclusively involves outpatient clinic visits. The app serves as a substitute for outpatient clinic visits for patients with COPD, while patients with asthma use it as a complement to these visits.

Patients eligible for enrollment in telemonitoring include those under treatment by a pulmonologist, with a history of COPD or symptomatic asthma, and who are digitally skilled, as determined by the nurse practitioner. Patients are given the choice to either accept or decline the app usage. This inclusion process, along with the adoption of the app, occurs approximately 6 weeks after diagnoses.

Within the app, patients are asked to complete the Clinical COPD Questionnaire or the Asthma Control Questionnaire on a weekly basis, transitioning to a monthly schedule later on. If the questionnaire score exceeds the predetermined critical value, a nurse initiates contact with the patient to investigate the reason for deterioration and determine appropriate interventions, such as adjustments to medication.

Adherence to the intervention is monitored through the completion of the required questionnaire in the telemonitoring system. If a patient repeatedly does not complete the questionnaires, the nurse specialist is notified and the telemonitoring process is discontinued for that patient. A detailed description of the intervention can be found in Multimedia Appendix 1.

### Data Collection

Patient characteristics and clinical outcomes were collected from medical records using Ctcue (IQVIA) [31]. The patient characteristics and clinical outcomes used as study outcomes were selected through literature review and consultations with health care experts [32-34]. The following patient characteristics were selected: gender, age, BMI, smoking status, home status, distance to hospital, place of residence, Charlson Comorbidity Index (CCI), Global Initiative for Chronic Obstructive Lung Disease (GOLD) stage, and predicted forced expiratory volume (FEV% pre). The Diagnosis Treatment Combination (DTC) was used to categorize patients based on their primary diagnosis of asthma or COPD. Selected clinical outcomes were hospitalizations related to COPD or asthma, ED visits related to COPD or asthma, and moderate exacerbations and outpatient clinic visits related to COPD or asthma, all expressed in number per person-year. Moderate exacerbations are defined as detected exacerbation leading to prescribed antibiotics or prednisone, without hospitalization or ED visit.

The CDMF (Claims-based, Disease-specific refinements, Matching translation to *ICD-10* [*International Statistical Classification of Diseases, Tenth Revision*], Flexibility to allow use as a chart review instrument) CCI coding scheme was used in this study to score the comorbidity index [35]. This coding scheme is an updated version of the CCI coding scheme, translated to match diagnoses according to the* ICD-10* system. It comprises 19 comorbidities and uses a weighted index that considers both the number and the severity of these conditions. The overall core is determined by a sum of the weights, with elevated scores indicating more severe comorbid conditions and a greater mortality risk [35,36].

### Ethical Considerations

The study was approved by the local feasibility committee of Isala Hospital, and a non-WMO declaration (ie, not subject to the Dutch Medical Research Involving Human Subjects Act) was granted (study number 20240117). Data acquisition followed the rules and procedures according to the General Data Protection Regulation [37]. All identifying data were pseudonymized by the CTcue data program and stored securely. Informed consent was not applicable as this was an observational study for which obtaining individual patient consent was not feasible. Patients who withheld consent in their electronic health record for the use of their data in scientific research were excluded from the study.

### Statistical Analysis

To ensure an adequate sample size for meaningful analysis of the outcomes, all patients within a 1-year time frame were included. This approach captures potential seasonal variations and a diverse range of patient cases, thereby enhancing the generalizability of findings.

Statistical analysis was performed using SPSS (version 26; IBM Corp). To describe patient characteristics, descriptive statistics were used. Categorical variables were presented using absolute and relative frequencies. Normally distributed continuous variables were presented using mean and SD, and nonnormally distributed continuous variables were presented using median and IQR. Group comparisons between the telemonitoring and conventional care groups were performed using statistical tests; chi-square test for categorical variables, 2-tailed student *t* test for normally distributed variables, and Mann-Whitney *U* test for nonnormally distributed variables. *P* values of <.05 were considered significant. Normality was checked with QQ-plots, histograms, and the Kolmogorov-Smirnov test.

To provide a comprehensive overview of clinical outcomes, both unadjusted and adjusted outcomes were analyzed. Including the unadjusted outcomes offers a transparent view of the direct effects of intervention use in clinical practice on observed differences, prior to adjustment for potential confounders. To describe clinical outcomes, incidence rates were used. Incidence rates for both groups were computed by dividing the number of events within that group by the total person-years for that group. Poisson regression was initially applied to estimate the incidence rate ratios (IRRs) with 95% CI. However, overdispersion was identified in the data, except for ED visits, which were equidispersed and suitable for Poisson regression. For all other clinical outcomes, negative binomial regression was used to account for overdispersion, providing IRRs, CIs, and *P* values. A *P* value of <.05 was considered significant. Given the differing care pathways for COPD and asthma, and the uneven distribution of these patients within the sample, subanalyses were conducted for patients with COPD and asthma. Age, gender, comorbidity burden, DTC, and smoking status are known confounders and found to be significantly different in the telemonitoring and conventional care groups and therefore included in the regression model [38-40].

When missing data occurred, the researcher tried to extract this information from the electronic patient dossier. Missing value patterns were observed, and the Little MCAR test was performed to test whether the missing data occurred completely at random. Multiple imputation with 10 imputed datasets was performed for all missing data, using the AUTO method to determine the most appropriate imputation technique based on the characteristics of the variables. Discrepancies between the datasets were checked. The bar procedure was used to pool all imputed data into 1 dataset [41].

## Results

### Missing Data

A total of 710 patients were included in the study; 614 in the conventional care group and 96 in the telemonitoring group. A total of 2.2% of missing data were identified from 5 variables. The variables smoking status, BMI, and GOLD stage displayed missing data rates below 5%. The variable FEV1% pre has a missing data percentage of 11.8% and home status percentage of 22.4% (Multimedia Appendix 2). Little MCAR test came out highly significantly at<0.0001, so the data were considered to be missing at random.

### Patient Characteristics

A balanced distribution of females and males was observed in the conventional care group (340/614, 55% female), compared with a higher proportion of females in the telemonitoring group (67/96, 70% female; Table 1). A significant age difference was found between the groups with a mean of 63 (IQR 48.00‐73.00) years in the conventional care group and a mean of 53 (IQR 41.00‐64.75) years in the telemonitoring group (*P*<.001). The prevalence of patients with asthma was higher in the telemonitoring group (74/96, 77%) than in the conventional care group (366/614, 60%) (*P*=.001). No patients with both asthma and COPD were observed. Smoking status differed substantially between the groups, mostly caused by a higher proportion of current smokers in the conventional care group (195/614, 32% current smokers) than in the telemonitoring group (10/96, 10% current smokers) (*P*<.001). The conventional care group showed more comorbidities (median 1.00, IQR 1.00‐3.00) than the telemonitoring group (median 1.00, IQR 1.00‐2.00) (*P*=.003).

In the subanalyses, differences in gender and age among patients with COPD were no longer observed (Multimedia Appendix 3). The CCI for patients with COPD was generally higher than that for patients with asthma, but no difference was found between the conventional care and telemonitoring groups within the COPD group. For patients with asthma, the CCI was no longer significantly different between the groups in subanalyses.

**Table 1.** Patient characteristics.

Characteristic	Telemonitoring (n=96)	Conventional care (n=614)	*P* value
Sex, n (%)			.01
Male	29 (30)	274 (45)	
Female	67 (70)	340 (55)	
Age (years), median (IQR)	53 (41.00‐64.75)	63 (48.00‐73.00)	<.001
BMI (kg/m^2^), median (IQR)	27.98 (24.98‐32.12)	26.70 (23.50‐30.69)	.12
Smoking status, n (%)			<.001
Never	45 (47)	190 (31)	
Former	41 (43)	229 (37)	
Current	10 (10)	195 (32)	
Home status, n (%)			.10
Living alone	17 (18)	156 (25)	
Living together	79 (82)	458 (75)	
Distance to hospital (km), median (IQR)	23.05 (10.48‐32.71)	21.18 (11.25‐30.98)	.60
Place of residence, n (%)			.82
Urban	50 (52)	312 (51)	
Rural	46 (48)	302 (49)	
Charlson Comorbidity Index (points), median (IQR)	1.00 (1.00‐2.00)	1.00 (1.00‐3.00)	.003
COPD^a^ GOLD^b^, n (%)			.85
GOLD I	1 (5)	11 (4)	
GOLD II	8 (36)	106 (43)	
GOLD III	10 (45)	110 (44)	
GOLD IV	3 (14)	21 (9)	
FEV1 % pre^c^, median (IQR)	81.50 (55.98‐97.80)	73.00 (54.00‐90.00)	.07
DTC^d^, n (%)			.001
Asthma	74 (77)	366 (60)	
COPD	22 (23)	248 (40)	

a COPD: chronic obstructive pulmonary disease.

b GOLD: Chronic Obstructive Lung Disease staging.

c FEV1 % pre: predicted forced expiratory volume in 1 second (%).

d DTC: Diagnosis Treatment Combination.

### Clinical Outcomes

#### Hospitalizations

A total of 46 hospitalizations related to COPD or asthma were recorded in the conventional care group, compared with 2 hospitalizations in the telemonitoring group. The total follow-up time in the conventional care group was 161,137 days, with a mean follow-up of 262 days. In the telemonitoring group, the total follow-up time was 30,087 days, with a mean follow-up of 313 days. This resulted in a hospitalization rate of 0.1 hospitalizations per person-year in the conventional care group, while the telemonitoring group had a lower hospitalization rate of 0.02.

Univariate analysis indicated that this difference was significant (IRR 0.24, 95% CI 0.06‐0.98; *P*=.047; Table 2). However, after adjusting for potential confounders, the difference between both groups due to telemonitoring was no longer significant (IRR 0.68, 95% CI 0.15‐3.11; *P*=.62; Table 3). Age, gender, type of condition (DTC), and smoking status were found to be significant contributors to the model (Multimedia Appendix 2). In subanalyses, similar results were found among patients with COPD. Among patients with asthma, the hospitalization rates were too low to perform a regression analysis, with a hospitalization rate of 0.02 in the conventional care group and zero in the telemonitoring group (Multimedia Appendix 3).

**Table 2.** Clinical outcomes.

Clinical outcome	Telemonitoring IR^a^ (number per person-year)	Conventional care IR (number per person-year)	IRR^b^ (95% CI)	*P* value
Hospitalizations^c^	0.02	0.1	0.24 (0.06‐0.98)	.047
Emergency department visits^c^	0.01	0.02	0.54 (0.07‐4.18)	.55
Moderate exacerbations^d^	0.22	0.13	1.67 (0.94‐2.96)	.08
Total outpatient consultations^c^	6.51	1.93	3.43 (2.69‐4.36)	<.001
Outpatient clinic visits^c^	1.33	1.18	1.11 (0.82‐1.49)	.50
Telephone and screen-to-screen consultations^c^	5.17	0.75	7.08 (5.46‐9.18)	<.001

a IR: incidence rate.

b IRR: incidence rate ratio.

c Related to chronic obstructive pulmonary disease or asthma.

d Prescribed antibiotics or prednisone for exacerbation not resulting in emergency department visit or hospitalization.

**Table 3.** Clinical outcomes adjusted for possible confounders^a^.

Clinical outcome	Telemonitoring IR^b^ (number per person-year)	Conventional care IR (number per person-year)	IRR^c^ (95% CI)	*P* value
Hospitalizations^d^	0.02	0.1	0.68 (0.15‐3.11)	.62
Emergency department visits^d^	0.01	0.02	0.90 (0.11‐7.50)	.92
Moderate exacerbations^e^	0.22	0.13	2.15 (1.16‐3.98)	.02
Total outpatient consultations^d^	6.51	1.93	3.51 (2.74‐4.50)	<.001
Outpatient clinic visits^d^	1.33	1.18	1.19 (0.88‐1.62)	.27
Telephone and screen-to-screen consultations^d^	5.17	0.75	7.16 (5.47‐9.36)	<.001

a Confounders: age, gender, comorbidity burden, Diagnosis Treatment Combination, and smoking status.

b IR: incidence rate.

c IRR: incidence rate ratio.

d Related to chronic obstructive pulmonary disease or asthma.

e Prescribed antibiotics or prednisone for exacerbation not resulting in emergency department visit or hospitalization.

#### ED Visits

A total of 10 ED visits related to COPD or asthma were recorded in the conventional care group, compared with only 1 in the telemonitoring group. This resulted in an ED visit rate of 0.02 ED visits per person-year in the conventional group and a lower rate of 0.01 in the telemonitoring group. Univariate analysis showed that this difference was not significant (IRR 0.54, 95% CI 0.07‐4.18; *P*=.55; Table 2). After adjusting for potential confounders, the result remained nonsignificant (IRR 0.90, 95% CI 0.11‐7.50; *P*=.92; Table 3). No other variables were found to be a significant contributor to the model (Multimedia Appendix 2). In subanalyses, no difference was found between the 2 groups among patients with COPD. The ED visit rates among patients with asthma were too low to perform a regression analysis, with an ED visit rate of 0.02 in the conventional care group and zero in the telemonitoring group (Multimedia Appendix 3).

#### Moderate Exacerbations

A total of 59 moderate exacerbations related to COPD or asthma were reported in the conventional care group, compared with 18 in the telemonitoring group. This resulted in an incidence rate of 0.13 exacerbations per person-year in the conventional group and a higher rate of 0.22 in the telemonitoring group. Univariate analysis showed no significant difference between the 2 groups (IRR 1.67, 95% CI 0.94‐2.96; *P*=.08; Table 2). However, after adjusting for possible confounders, moderate exacerbations were found to be significantly different, with fewer exacerbations in the conventional care group (IRR 2.15, 95% CI 1.16‐3.98; *P*=.02; Table 3).

The type of condition was also found to be a significant contributor to the model (Multimedia Appendix 2). In subanalyses, a significant difference was initially found between the 2 groups among patients with COPD, but this effect disappeared after correcting for potential confounders. No difference was observed between the 2 groups among patients with asthma (Multimedia Appendix 3).

#### Outpatient Clinic Visits

A total of 853 outpatient clinic visits and telephone and screen-to-screen consultations related to COPD or asthma were reported in the conventional care group, compared with 537 in the telemonitoring group. This resulted in an incidence rate of 1.93 in the conventional care group and a considerably higher rate of 6.51 in the telemonitoring group. This difference was significant in univariate analysis (IRR 3.43, 95% CI 2.69‐4.36; *P*<.001; Table 2). When separated into outpatient clinic visits and telephone and screen-to-screen consultations, a significant difference was found only in the telephone and screen consultations (IRR 7.08, 95% CI 5.46‐9.18; *P*<.001). After adjusting for potential confounders, these outcomes remained similar (Table 3). No other variables were found to be a significant contributor to the model for outpatient clinic visits (Multimedia Appendix 2). Subanalyses for COPD and asthma yielded results consistent with the main analyses (Multimedia Appendix 3).

## Discussion

### Principal Results

We compared the characteristics and clinical outcomes of patients receiving telemonitoring versus those receiving conventional care, noting that a relatively small group of patients was in the telemonitoring group (14%) compared with the conventional care group (86%). We found some significant differences between the characteristics of both groups. Patients with COPD or asthma using telemonitoring tend to be younger than those receiving conventional care, predominantly female, less likely to be current smokers, and have fewer comorbidities. Patients with asthma tend to use telemonitoring more than patients with COPD. Although the CCI was significantly different in overall analyses, this difference disappeared in the subgroup analysis. This change appears to be due to the higher proportion of patients with asthma in the telemonitoring group, who generally have a lower CCI.

Considering clinical outcomes, we found some remarkable results. Patients using telemonitoring showed a higher frequency of exacerbations treated with antibiotics or prednisone, but no significant difference was found in hospitalizations and ED visits. Furthermore, patients using telemonitoring had significantly more telephone and screen-to-screen consultations, but outpatient clinic visits remained consistent across both groups.

### Comparison With Prior Work

The tendency for younger patients to use telemonitoring more is consistent with findings in the literature. Age above 65 years is widely seen as a predictor of lower eHealth usage [32,42]. This trend is likely due to factors such as lower digital skills, lack of interest in digital apps, cognitive impairments, or the absence of a caregiver to assist with technology [43,44]. Additionally, health care providers may be reluctant to offer telemonitoring to older patients when they perceive lower confidence in the patient’s ability to use digital tools [45]. Studies have recommended that telemonitoring apps should be adapted to meet the needs of older adults by incorporating interactive functionalities or by employing community health workers to assist the older adults with digital apps [43,46].

More females than males were included in telemonitoring, which is consistent with findings in the literature, although no specific underlying reason could be identified [32,34]. This higher inclusion of females was observed only among patients with asthma. Among patients with COPD, the gender distribution was equal. Therefore, the observed gender difference in the overall analysis was attributable solely to the patients with asthma.

When comparing both patient populations, more patients with asthma used telemonitoring than patients with COPD. This may partially stem from the asthma app being complementary to conventional care, while the COPD app substitutes for it. Patients might be reluctant to use telemonitoring as a substitute for attending the outpatient clinic, as they are used to receiving face-to-face care. Additionally, patients with asthma, being generally younger and having fewer comorbidities than those with COPD, are consequently more inclined to engage with telemonitoring.

A higher frequency of exacerbations treated with antibiotics or prednisone was found in the telemonitoring group. No other studies were found in the literature with this outcome measure. This higher frequency might be attributed to the early recognition of deterioration and easily accessible contact with health care providers through the app. With these early interventions, the objective is to prevent ED visits and hospitalizations. However, despite observing a reduction in hospitalizations and ED visits, this was not significant after adjusting for confounders. The evidence in the literature on this topic is mixed. For patients with asthma, systematic reviews were found indicating no reduction in ED visits [47] but a significant reduction in hospitalization, especially for patients with more severe asthma [16,47]. Other studies have reported conflicting evidence [48]. For patients with COPD, some systematic reviews found a significant reduction in ED visits [22,49] and hospitalizations [22,23,49], while others reported conflicting or no significant effects [20,21,24]. Interestingly, in patients with both COPD and asthma, evidence was found that longer use of telemonitoring (12 months or longer) was more effective in reducing hospitalization [16,22].

Utilization of telemonitoring did not result in fewer outpatient clinic visits, despite one of the app’s aims being to reduce such visits [50]. This may be attributed to health care providers continuing to schedule outpatient clinic visits alongside telemonitoring, driven by their sense of responsibility for their patients’ health or by the lack of clear integration of telemonitoring into routine care pathways [45]. Patients using telemonitoring have considerably more telephone and screen-to-screen consultations. The increase in these types of consultations, along with more prescribed antibiotics or prednisone and no reduction in hospitalizations and ED visits, might indicate that telemonitoring is not cost-effective as overall health care consumption increases. This is in line with studies reporting no or uncertain effect on cost-effectiveness [24,51]. However, the focus should not solely be on reducing health care costs but also on its ability to enhance access to care and improved disease management and quality of life [17,18,52]. Therefore, discussions about the implementation of telemonitoring should not be limited to costs but also improvements in the accessibility and quality of care and patient and health care professionals’ perspectives.

### Limitations

The results of our study should be interpreted in the light of some limitations. First, this study used real-world data, which tend to be unstructured and inconsistent due to variations in data entry methods among health care providers [27]. Not all required data for this study were found documented, such as educational level and socioeconomic status, although these variables can be associated with digital use [32,34]. This study did not include additional sensitivity analyses to assess residual confounding. While known confounders that were documented were controlled for, the potential impact of unmeasured factors cannot be ruled out. Missing data posed a challenge. Global initiative for asthma stages for assessing asthma severity could not be used due to a significant amount of missing data; FEV1% pre was used as an alternative measure. Two variables used showed a missing data rate above 10%, which may lead to bias in results [53]. To address this issue, multiple imputation was used, and all imputed data were examined. No discrepancies were found in the distribution or significance between the imputed datasets. Also, information about causes for hospitalization, ED visits, and prescribing antibiotics of prednisone was not always clearly documented. To overcome this problem, all lung-related hospitalizations, ED visits, and prescribed antibiotics and prednisone were checked by the first author in the electronic patient dossier. Furthermore, the use of purely CCI scores for considering comorbidities may not have captured all relevant conditions; a more detailed consideration of these comorbidities would have provided a firmer description of the population in this study.

Second, this study is a single-center study and therefore not immediately transferrable to other hospitals. Nevertheless, this study was conducted within a hospital with extensive expertise in telemonitoring and therefore offers valuable insights that can serve as guidance for health care institutions implementing telemonitoring interventions. The app used in this study is widely implemented in hospitals across the Netherlands. Although this was a single-site study, the widespread use of the app strengthens the potential generalizability of the results to similar clinical contexts.

Third, although the sample size provided sufficient insight into differences in patient characteristics and clinical outcomes, the low prevalence of the clinical outcomes, particularly in the smaller telemonitoring group, limits the statistical power and reduces the sensitivity to detect true differences between the 2 groups. While a larger sample size might potentially reveal differences in hospitalization rates and ED visits, their low prevalence diminishes their clinical relevance. Additionally, due to the low rates of hospitalization and ED visits among patients with asthma in the telemonitoring group, no differences could be calculated in this subgroup analysis. Nonetheless, the data from this study can be used to pool with other datasets to draw firmer conclusions.

In this study, we aimed to explore the differences in characteristics and clinical outcomes between patients using telemonitoring and those receiving conventional care in a real-world setting, where patients could choose to accept or decline the app based on their preferences. This approach may introduce self-selection bias, which could affect the comparability of the groups. However, rather than mitigate this bias, this study aimed to embrace it, as it offers valuable insights into how telemonitoring performs when patients voluntarily engage with it.

### Recommendations for Clinical Practice and Research

Understanding the characteristics of patients using telemonitoring is crucial for adapting the intervention to better suit current users and enhance accessibility for those who are not currently benefiting [46]. More insight is needed regarding the factors influencing lower adoption rates of telemonitoring among men, older adults, and nonsmokers. Hence, qualitative research is recommended to explore the considerations made by patients to either accept or decline telemonitoring, as well as the considerations health care providers take into account when deciding whether to enroll patients in telemonitoring.

Clinical outcomes in this study were assessed for a maximum duration of 1 year after the start of the intervention. Considering that one of the intervention’s objectives is to improve self-management and learning to recognize deterioration early, further research examining the long-term effects of telemonitoring is recommended, especially since evidence was found that positive effects might emerge later on [16,22]. Possibly, patients require an adjustment period for positive effects to manifest at a later stage. A longer study duration would not only allow for these delayed benefits to manifest but also provide more robust and valid results regarding clinical outcomes.

Patients often continue to attend outpatient clinic visits despite being enrolled in telemonitoring. To reduce health care usage, it is crucial to provide patients with additional guidance on effectively using the communication channels offered by telemonitoring. This may encourage them to use these methods instead of attending outpatient clinic visits. Additionally, when implementing telemonitoring, it is essential to critically evaluate and redesign care processes to prevent unnecessary health care use.

When evaluating the cost-effectiveness of telemonitoring, future research should not solely focus on reducing health care costs but should also emphasize the delivery of improved care at a reasonable cost [52]. High quality of care, which includes improved access, better disease management, and improved quality of life, may justify the financial investment in telemonitoring. In this context, user satisfaction and patient experience are also critical components of quality of care. Future studies should therefore incorporate user feedback to gain deeper insights into how telemonitoring is perceived by patients.

### Conclusions

Patient characteristic differences and clinical outcome differences were identified between telemonitoring and conventional care. Although telemonitoring facilitated earlier initiation of treatment, it did not lead to fewer hospital or outpatient clinic visits. More insight is needed into factors influencing participation in telemonitoring to better serve current users and to improve accessibility for nonusers. Patients should be provided with additional guidance on effectively using the communication channels offered by telemonitoring. This may encourage them to use these methods instead of attending outpatient clinic visits. Additionally, when implementing telemonitoring, it is essential to critically evaluate and redesign care processes to prevent unnecessary health care use.

## Supplementary material

10.2196/66743 Multimedia Appendix 1 Telemonitoring mobile app.

10.2196/66743 Multimedia Appendix 2 SPSS output.

10.2196/66743 Multimedia Appendix 3 Subanalyses of asthma or chronic obstructive pulmonary disease.

## Abbreviations

CCI: Charlson Comorbidity Index
CDMF: Claims-based, Disease-specific refinements, Matching translation to *ICD-10*, Flexibility to allow use as a chart review instrument
COPD: chronic obstructive pulmonary disease
DTC: Diagnosis Treatment Combination
ED: emergency department
FEV% pre: predicted forced expiratory volume
GOLD: Global Initiative for Chronic Obstructive Lung Disease
*ICD-10*: *International Statistical Classification of Diseases, Tenth Revision*
IRR: incidence rate ratio
RCT: randomized controlled trial
RWE: real-world evidence
STROBE: Strengthening the Reporting of Observational Studies in Epidemiology
