# Supplementary material for: Real-World Comparison of Telemonitoring Versus Conventional Care in Patients With Chronic Obstructive Pulmonary Disease and Those With Asthma—Impact on Clinical Outcomes and Patient Characteristics: Retrospective Cohort Study
Source: J Med Internet Res. 2025 Aug 14;27:e66743. doi: 10.2196/66743 (PMC12371767; doi:10.2196/66743)
Supplement: Multimedia Appendix 1 [file jmir-v27-e66743-s001.docx]

**Multimedia Appendix 1. Telemonitoring mobile application**

Inclusion criteria for enrollment in the COPD telemonitoring application:

- - Patient is under treatment by a pulmonologist
  - Patient has a history of COPD GOLD III, IV or II with high symptom burden class B
  - Patient has had at least two exacerbations in the past year or has self-management goals
  - Patient is competent in the Dutch language
  - Patient is digital skilled and has WiFi at home

Exclusion criteria:

- - Patient is in a terminal stage of their disease
  - Patient is actively participating in clinical rehabilitation
  - Patient is currently admitted with an exacerbation COPD
  - Patient is undergoing active treatment for a malignant condition

Inclusion criteria to get enrolled in the asthma telemonitoring application:

- - Patient has a history of symptomatic asthma
  - Diagnoses of asthma has been made by the treating pulmonologist
  - Patient is competent in the Dutch language
  - Patient is somewhat digital skilled

Exclusion criteria:

- - Patient is actively participating in clinical rehabilitation

The nurse practitioner determines which measurements patients should complete and when, and develops an asthma or COPD action plan. The critical value of the Asthma Control Questionnaire (ACQ) is initially set at 1.5 or adjusted as needed for the patient. For the Clinical COPD Questionnaire (CCQ), the nurse practitioner establishes a critical value together with the patient.

*Weekly phase*

Patients complete the ACQ or CCQ on a weekly basis the first eight weeks (asthma) or four months (COPD).^27,29^ If the patient experiences symptoms, the patient has the option to fill out the questionnaire more frequently. When the score on this questionnaire exceeds the predetermined critical value in the first eight weeks, a nurse initiates contact with the patient to investigate the reason for deterioration and determine necessary interventions, such as adjustments to medication. Three weeks after initiation of the application, the patient visits the nurse practitioner for an administrative checkup.

*Monthly phase*

After completing the first phase, the patients make a transition to a monthly schedule of filling in the ACQ or CCQ. This phase lasts for sixteen weeks (asthma) or five months (COPD).

After the transition, the nurse does not initiate contact if the score exceeds the critical value. Patients are able to request contact with a healthcare provider by using that option in the application.

*Self-management phase*

After completing the monthly phase, the patient transitions to the self-management phase where patients fill in the questionnaire according to preference. If needed, the patient can contact the healthcare provider by phone. After three months of self-management the patient is scheduled for an appointment for evaluation. If self-management is deemed sufficient, the patient gets referred back to primary care. Alternatively, if the patients asthma or COPD is deemed uncontrolled, or there are issues with self-management or significant comorbidities, the patient continues to receive treatment in secondary care.

*Alerts*

A red alert is sent to the healthcare professional when the ACQ/CCQ has been completed but exceeded the threshold value. A remark alert is sent when a patient has add a comment in the application. These comments can withhold a symptom update or a question about the disease. An overdue alert is sent when a patient fails to respond in time to a request for data or when a measurement or action is not completed within the designated time limit.
