# Supplementary material for: Real-World Comparison of Telemonitoring Versus Conventional Care in Patients With Chronic Obstructive Pulmonary Disease and Those With Asthma—Impact on Clinical Outcomes and Patient Characteristics: Retrospective Cohort Study
Source: J Med Internet Res. 2025 Aug 14;27:e66743. doi: 10.2196/66743 (PMC12371767; doi:10.2196/66743)
Supplement: Multimedia Appendix 2 [file jmir-v27-e66743-s002.docx]

**Multimedia Appendix 2. SPSS output.**

Missing values

## Variable Summary^a,b^

| Missing | | |  |  |  |
| --- | --- | --- | --- | --- | --- |
| N | | Percent | Valid N | Mean | Std. Deviation |
| Home_status | 159 | 22,4% | 551 |  |  |
| FEV1_proc_pred | 84 | 11,8% | 626 | 73,0937 | 28,70271 |
| Smoking_status | 24 | 3,4% | 686 |  |  |
| BMI | 22 | 3,1% | 688 | 27,6368 | 6,08678 |
| GOLD_Copy | 7 | 1,0% | 703 |  |  |

1. Maximum number of variables shown: 25
2. Minimum percentage of missing values for variable to be included: 0,0%

Mann Whitney U Distance to hospital, Age, BMI, FEV1 % pre, CCI

| **Statistics** | | | | | | | |
| --- | --- | --- | --- | --- | --- | --- | --- |
| Telemonitoring |  |  | Distance_to_hos  pitalm | Age | BMI | FEV1_proc_  pred | CCI |
| conventional care | N | Valid | 614 | 614 | 614 | 614 | 614 |
|  |  | Missi  ng | 0 | 0 | 0 | 0 | 0 |
|  | Median | | 21176,00 | 63,00 | 26,70  00 | 73,0000 | 1,00 |
|  | Percentil es | 25 | 11246,50 | 48,00 | 23,50  00 | 54,0000 | 1,00 |
|  |  | 50 | 21176,00 | 63,00 | 26,70  00 | 73,0000 | 1,00 |
|  |  | 75 | 30982,25 | 73,00 | 30,69  25 | 90,0000 | 3,00 |
| telemonitoring | N | Valid | 96 | 96 | 96 | 96 | 96 |
|  |  | Missi  ng | 0 | 0 | 0 | 0 | 0 |
|  | Median | 25 | 23049,00 | 53,00 | 27,97  50 | 81,5000 | 1,00 |
|  | Percentil es |  | 10478,25 | 41,00 | 24,98  25 | 55,9825 | 1,00 |
|  |  | 50 | 23049,00 | 53,00 | 27,97  50 | 81,5000 | 1,00 |
|  |  | 75 | 32710,75 | 64,75 | 32,12  25 | 97,7951 | 2,00 |

## Ranks

|  | | Telemonitoring N | | | | Mean  Rank | | Sum of  Ranks | |
| --- | --- | --- | --- | --- | --- | --- | --- | --- | --- |
| Distance_to_hospitalm | | conventional care | 614 | | | 353,88 | | 217283,50 | |
|  |  | telemonitoring | 96 | | | 365,85 | | 35121,50 | |
|  |  | Total | 710 | | |  | |  | |
| Age conventional care | | | 614 | | | 368,95 | | 226533,00 | |
|  | | telemonitoring | | | 96 | 269,50 | | 25872,00 | |
|  |  | Total | | | 710 |  | |  | |
| BMI | | conventional care | | | 614 | 350,80 | | 215388,50 | |
|  |  | telemonitoring | | | 96 | 385,59 | | 37016,50 | |
|  |  | Total | | | 710 |  | |  | |
| FEV1_proc_pred | | conventional care | | | 614 | 349,97 | | 214882,50 | |
|  |  | telemonitoring | | | 96 | 390,86 | | 37522,50 | |
|  |  | Total | | | 710 |  | |  | |
| CCI | | conventional care | | | 614 | 363,69 | | 223307,00 | |
|  |  | telemonitoring | | | 96 | 303,10 | | 29098,00 | |
|  |  | Total | | | 710 |  | |  | |

**Test Statistics^a^**

| Distance_to_hospit  alm | | Age | BMI | FEV1_proc_p  red | CCI |
| --- | --- | --- | --- | --- | --- |
| Mann-Whitney U | 28478,500 | 21216,00  0 | 26583,500 | 26077,500 | 24442,00  0 |
| Wilcoxon W | 217283,500 | 25872,00  0 | 215388,50  0 | 214882,500 | 29098,00  0 |
| Z | -,532 | -4,419 | -1,546 | -1,816 | -2,947 |
| Asymp. Sig. (2-  tailed) | ,595 | ,000 | ,122 | ,069 | ,003 |

1. Grouping Variable: Telemonitoring

Chi-square Place of residence

| **Crosstab** | | | | | |
| --- | --- | --- | --- | --- | --- |
| Place_of_residence | | | | | Total |
| urban | | | | Rural |  |
| Telemonitoring | conventional care | Count | 312 | 302 | 614 |
|  |  | Expected Count | 313,1 | 300,9 | 614,0 |
|  |  | % within Telemonitoring | 50,8% | 49,2% | 100,0% |
|  |  | % within  Place_of_residence | 86,2% | 86,8% | 86,5% |
|  |  | % of Total | 43,9% | 42,5% | 86,5% |
|  | telemonitoring | Count | 50 | 46 | 96 |
|  |  | Expected Count | 48,9 | 47,1 | 96,0 |
|  |  | % within Telemonitoring | 52,1% | 47,9% | 100,0% |
|  |  | % within  Place_of_residence | 13,8% | 13,2% | 13,5% |
|  |  | % of Total | 7,0% | 6,5% | 13,5% |
| Total | | Count | 362 | 348 | 710 |
|  |  | Expected Count | 362,0 | 348,0 | 710,0 |
|  |  | % within Telemonitoring | 51,0% | 49,0% | 100,0% |
|  |  | % within  Place_of_residence | 100,0% | 100,0% | 100,0% |
|  |  | % of Total | 51,0% | 49,0% | 100,0% |

## Chi-Square Tests

| Value | | df | Asymptotic Significance  (2-sided) | Exact Sig. (2- sided) | Exact Sig. (1- sided) |
| --- | --- | --- | --- | --- | --- |
| Pearson Chi-Square | ,053a | 1 | ,817 |  |  |
| Continuity Correctionb | ,015 | 1 | ,903 |  |  |
| Likelihood Ratio | ,054 | 1 | ,817 |  |  |
| Fisher's Exact Test |  |  |  | ,827 | ,452 |
| Linear-by-Linear  Association | ,053 | 1 | ,817 |  |  |
| N of Valid Cases | 710 |  |  |  |  |

1. 0 cells (0,0%) have expected count less than 5. The minimum expected count is 47,05.
2. Computed only for a 2x2 table

Chi-square Gender

| **Crosstab** | | | | | |
| --- | --- | --- | --- | --- | --- |
| Gender | | | | | Total |
| male | | | | female |  |
| Telemonitoring | conventional care | Count | 274 | 340 | 614 |
|  |  | Expected Count | 262,0 | 352,0 | 614,0 |
|  |  | % within Telemonitoring | 44,6% | 55,4% | 100,0% |
|  |  | % within Gender | 90,4% | 83,5% | 86,5% |
|  |  | % of Total | 38,6% | 47,9% | 86,5% |
|  | telemonitoring | Count | 29 | 67 | 96 |
|  |  |  |  |  |  |
|  |  | Expected Count | 41,0 | 55,0 | 96,0 |
|  |  | % within Telemonitoring | 30,2% | 69,8% | 100,0% |
|  |  | % within Gender | 9,6% | 16,5% | 13,5% |
|  |  | % of Total | 4,1% | 9,4% | 13,5% |
| Total | | Count | 303 | 407 | 710 |
|  |  | Expected Count | 303,0 | 407,0 | 710,0 |
|  |  | % within Telemonitoring | 42,7% | 57,3% | 100,0% |
|  |  | % within Gender | 100,0% | 100,0% | 100,0% |
|  |  | % of Total | 42,7% | 57,3% | 100,0% |

## Chi-Square Tests

| Value | | df | Asymptotic  Significance (2-sided) | Exact Sig. (2- sided) | Exact Sig. (1- sided) |
| --- | --- | --- | --- | --- | --- |
| Pearson Chi-Square | 7,054a | 1 | ,008 |  |  |
| Continuity Correctionb | 6,477 | 1 | ,011 |  |  |
| Likelihood Ratio | 7,281 | 1 | ,007 |  |  |
| Fisher's Exact Test |  |  |  | ,008 | ,005 |
| Linear-by-Linear  Association | 7,044 | 1 | ,008 |  |  |
| N of Valid Cases | 710 |  |  |  |  |

1. 0 cells (0,0%) have expected count less than 5. The minimum expected count is 40,97.
2. Computed only for a 2x2 table

Chi-square DTC

| **Crosstab** | | | | | |
| --- | --- | --- | --- | --- | --- |
|  | | | | | Total |
| Asthma | | | | COPD |  |
| Telemonitoring | conventional care | Count | 366 | 248 | 614 |
|  |  | Expected Count | 380,5 | 233,5 | 614,0 |
|  |  | % within Telemonitoring | 59,6% | 40,4% | 100,0% |
|  |  | % within DBC | 83,2% | 91,9% | 86,5% |
|  |  | % of Total | 51,5% | 34,9% | 86,5% |
|  | telemonitoring | Count | 74 | 22 | 96 |
|  |  | Expected Count | 59,5 | 36,5 | 96,0 |
|  |  | % within Telemonitoring | 77,1% | 22,9% | 100,0% |
|  |  | % within DBC | 16,8% | 8,1% | 13,5% |
|  |  | % of Total | 10,4% | 3,1% | 13,5% |
| Total | | Count | 440 | 270 | 710 |
|  |  | Expected Count | 440,0 | 270,0 | 710,0 |
|  |  | % within Telemonitoring | 62,0% | 38,0% | 100,0% |
|  |  | % within DBC | 100,0% | 100,0% | 100,0% |
|  |  | % of Total | 62,0% | 38,0% | 100,0% |

## Chi-Square Tests

| Value | | df | Asymptotic Significance  (2-sided) | Exact Sig. (2- sided) | Exact Sig. (1- sided) |
| --- | --- | --- | --- | --- | --- |
| Pearson Chi-Square | 10,757a | 1 | ,001 |  |  |
| Continuity Correctionb | 10,028 | 1 | ,002 |  |  |
| Likelihood Ratio | 11,454 | 1 | ,001 |  |  |
| Fisher's Exact Test |  |  |  | ,001 | ,001 |
| Linear-by-Linear  Association | 10,741 | 1 | ,001 |  |  |
| N of Valid Cases | 710 |  |  |  |  |

1. 0 cells (0,0%) have expected count less than 5. The minimum expected count is 36,51.
2. Computed only for a 2x2 table

Chi-square Smoking status

| **Crosstab** |  |
| --- | --- |
| Smoking_status | Total |

| Never | | | | Former | Current |  |
| --- | --- | --- | --- | --- | --- | --- |
| Telemonitorin g | conventional care | Count | 190 | 229 | 195 | 614 |
|  |  | Expected Count | 203,2 | 233,5 | 177,3 | 614,0 |
|  |  | % within  Telemonitoring | 30,9% | 37,3% | 31,8% | 100,0  % |
|  |  | % within  Smoking_status | 80,9% | 84,8% | 95,1% | 86,5% |
|  |  | % of Total | 26,8% | 32,3% | 27,5% | 86,5% |
|  | telemonitoring | Count | 45 | 41 | 10 | 96 |
|  |  | Expected Count | 31,8 | 36,5 | 27,7 | 96,0 |
|  |  | % within  Telemonitoring | 46,9% | 42,7% | 10,4% | 100,0  % |
|  |  | % within  Smoking_status | 19,1% | 15,2% | 4,9% | 13,5% |
|  |  | % of Total | 6,3% | 5,8% | 1,4% | 13,5% |
| Total | | Count | 235 | 270 | 205 | 710 |
|  |  | Expected Count | 235,0 | 270,0 | 205,0 | 710,0 |
|  |  | % within  Telemonitoring | 33,1% | 38,0% | 28,9% | 100,0  % |
|  |  | % within  Smoking_status | 100,0  % | 100,0  % | 100,0  % | 100,0  % |
|  |  | % of Total | 33,1% | 38,0% | 28,9% | 100,0  % |

## Chi-Square Tests

| Value | | df | Asymptotic Significance (2-  sided) |
| --- | --- | --- | --- |
| Pearson Chi-Square | 20,102^a^ | 2 | ,000 |
| Likelihood Ratio | 23,128 | 2 | ,000 |
| Linear-by-Linear Association | 18,638 | 1 | ,000 |
| N of Valid Cases | 710 |  |  |

1. 0 cells (0,0%) have expected count less than 5. The minimum expected count is 27,72.

Post hoc analysis Smoking status

## Telemonitoring * Smoking_status Crosstabulation

| Smoking_status | | | | | | Total |
| --- | --- | --- | --- | --- | --- | --- |
| Never | | | | Former | Current |  |
| Telemonitorin g | conventional care | Count | 190 | 229 | 195 | 614 |
|  |  | % within  Telemonitoring | 30,9% | 37,3% | 31,8% | 100,0% |
|  |  | Adjusted Residual | -3,1 | -1,0 | 4,3 |  |
|  | telemonitoring | Count | 45 | 41 | 10 | 96 |
|  |  | % within  Telemonitoring | 46,9% | 42,7% | 10,4% | 100,0% |
|  |  | Adjusted Residual | 3,1 | 1,0 | -4,3 |  |
| Total | | Count | 235 | 270 | 205 | 710 |
|  |  | % within  Telemonitoring | 33,1% | 38,0% | 28,9% | 100,0% |

Chi-square Home status

| **Crosstab** | | | | | |
| --- | --- | --- | --- | --- | --- |
| Home_status | | | | | Total |
| Living  alone | | | | Living  together |  |
| Telemonitorin g | conventional care | Count | 156 | 458 | 614 |
|  |  | Expected Count | 149,6 | 464,4 | 614,0 |
|  |  | % within  Telemonitoring | 25,4% | 74,6% | 100,0% |
|  |  | % within Home_status | 90,2% | 85,3% | 86,5% |
|  |  | % of Total | 22,0% | 64,5% | 86,5% |
|  | telemonitoring | Count | 17 | 79 | 96 |
|  |  | Expected Count | 23,4 | 72,6 | 96,0 |
|  |  | % within  Telemonitoring | 17,7% | 82,3% | 100,0% |
|  |  | % within Home_status | 9,8% | 14,7% | 13,5% |
|  |  | % of Total | 2,4% | 11,1% | 13,5% |
| Total | | Count | 173 | 537 | 710 |
|  |  | Expected Count | 173,0 | 537,0 | 710,0 |
|  |  | % within  Telemonitoring | 24,4% | 75,6% | 100,0% |
|  |  | % within Home_status | 100,0% | 100,0% | 100,0% |
|  |  | % of Total | 24,4% | 75,6% | 100,0% |

## Chi-Square Tests

| Value | | df | Asymptotic Significance  (2-sided) | Exact Sig. (2- sided) | Exact Sig. (1- sided) |
| --- | --- | --- | --- | --- | --- |
| Pearson Chi-Square | 2,670a | 1 | ,102 |  |  |
| Continuity Correctionb | 2,269 | 1 | ,132 |  |  |
| Likelihood Ratio | 2,836 | 1 | ,092 |  |  |
| Fisher's Exact Test |  |  |  | ,124 | ,063 |
| Linear-by-Linear  Association | 2,666 | 1 | ,102 |  |  |
| N of Valid Cases | 710 |  |  |  |  |

1. 0 cells (0,0%) have expected count less than 5. The minimum expected count is 23,39.
2. Computed only for a 2x2 table

Univariate negative binomial regression - hospitalizations

| **Parameter Estimates** | | | | | | |
| --- | --- | --- | --- | --- | --- | --- |
| Parameter | B | Std. Err or | 95% Wald Confidence Interval | Hypothesis Test | Ex p(B  ) | 95% Wald Confidence Interval for Exp(B) |

|  | |  | Lower | Upper | Wald  Chi- Square | df | Sig  . |  | Lower | Upper |
| --- | --- | --- | --- | --- | --- | --- | --- | --- | --- | --- |
| (Intercept) | -  8,1  67 | ,15  31 | -8,467 | -7,867 | 2847,0  72 | 1 | ,00  0 | ,00  0 | ,000 | ,000 |
| Telemonito ring | -  1,4  49 | ,73  09 | -2,881 | -,016 | 3,929 | 1 | ,04  7 | ,23  5 | ,056 | ,984 |
| (Scale) | 1a |  |  |  |  |  |  |  |  |  |
| (Negative  binomial) | 1a |  |  |  |  |  |  |  |  |  |

Dependent Variable: Hospitalizations

Model: (Intercept), Telemonitoring, offset = ln_followup_time

1. Fixed at the displayed value.

Univariate Poisson regression - ED visits

## Parameter Estimates

| 95% Wald  Confidence Interval | | | | | Hypothesis Test | | | 95% Wald  Confidence Interval for Exp(B) | | |
| --- | --- | --- | --- | --- | --- | --- | --- | --- | --- | --- |
| Parameter | B | Std. Erro  r | Lower | Upper | Wald Chi- Squar  e | df | Sig  . | Exp( B) | Lower | Upper |
| (Intercept) | -  9,6  87 | ,316  2 | - 10,30  7 | -9,068 | 938,46  2 | 1 | ,00  0 | 6,20  6E-5 | 3,339  E-5 | ,000 |
| Telemonitori ng | -  ,62  4 | 1,04  88 | -2,680 | 1,431 | ,354 | 1 | ,55  2 | ,536 | ,069 | 4,184 |
| (Scale) | 1a |  |  |  |  |  |  |  |  |  |

Dependent Variable: ED_visits

Model: (Intercept), Telemonitoring, offset = ln_followup_time

1. Fixed at the displayed value.

Univariate negative binomial regression - Total outpatient consultations

## Parameter Estimates

| 95% Wald Confidence  Interval | | | | | Hypothesis Test | | | 95% Wald Confidence  Interval for Exp(B) | | |
| --- | --- | --- | --- | --- | --- | --- | --- | --- | --- | --- |
| Parameter | B | Std. Err  or | Lower | Upper | Wald Chi-  Square | df | Sig  . | Ex p(B  ) | Lower | Upper |
| (Intercept) | -  5,2  37 | ,05  32 | -5,341 | -5,133 | 9701,2  82 | 1 | ,00  0 | ,00  5 | ,005 | ,006 |
| Telemonito  ring | 1,2  32 | ,12  32 | ,990 | 1,473 | 100,00  8 | 1 | ,00  0 | 3,4  28 | 2,693 | 4,364 |
| (Scale) | 1a |  |  |  |  |  |  |  |  |  |
| (Negative  binomial) | 1a |  |  |  |  |  |  |  |  |  |

Dependent Variable: Outpatient_and_telephone

| Model: (Intercept), Telemonitoring, offset = ln_followup_time |
| --- |
| a. Fixed at the displayed value. |

Univariate negative binomial regression - Outpatient clinic visits

## Parameter Estimates

| 95% Wald Confidence  Interval | | | | | Hypothesis Test | | | 95% Wald Confidence  Interval for Exp(B) | | |
| --- | --- | --- | --- | --- | --- | --- | --- | --- | --- | --- |
| Parameter | B | Std.  Err or | Lower | Upper | Wald  Chi- Square | df | Sig  . | Ex p(B  ) | Lower | Upper |
| (Intercept) | -  5,7  33 | ,05  98 | -5,850 | -5,616 | 9192,5  81 | 1 | ,00  0 | ,00  3 | ,003 | ,004 |
| Telemonito  ring | ,10  2 | ,15  25 | -,197 | ,401 | ,446 | 1 | ,50  4 | 1,1  07 | ,821 | 1,493 |
| (Scale) | 1a |  |  |  |  |  |  |  |  |  |
| (Negative  binomial) | 1a |  |  |  |  |  |  |  |  |  |

Dependent Variable: Outpatient_clinic_visits

Model: (Intercept), Telemonitoring, offset = ln_followup_time

a. Fixed at the displayed value.

Univariate negative binomial regression - Telephone and screen-to-screen consultations

## Parameter Estimates

| 95% Wald Confidence  Interval | | | | | Hypothesis Test | | | 95% Wald Confidence  Interval for Exp(B) | | |
| --- | --- | --- | --- | --- | --- | --- | --- | --- | --- | --- |
| Parameter | B | Std. Err  or | Lower | Upper | Wald Chi-  Square | df | Sig  . | Ex p(B  ) | Lower | Upper |
| (Intercept) | -  6,1  81 | ,06  85 | -6,315 | -6,047 | 8131,8  11 | 1 | ,00  0 | ,00  2 | ,002 | ,002 |
| Telemonito  ring | 1,9  57 | ,13  25 | 1,697 | 2,217 | 218,21  8 | 1 | ,00  0 | 7,0  77 | 5,459 | 9,175 |
| (Scale) | 1a |  |  |  |  |  |  |  |  |  |
| (Negative  binomial) | 1a |  |  |  |  |  |  |  |  |  |

Dependent Variable: Telephone_consultation

Model: (Intercept), Telemonitoring, offset = ln_followup_time

a. Fixed at the displayed value.

Negative binomial regression - hospitalizations with confounders

|  | | | | **Parameter Estimates** | | |  | | |
| --- | --- | --- | --- | --- | --- | --- | --- | --- | --- |
|  |  |  | 95% Wald Confidence  Interval | | Hypothesis Test | | |  | 95% Wald Confidence Interval for  Exp(B) |
| Para  meter | B | Std.  Error | Lowe  r | Uppe  r | Wald  Chi- | df | Sig. | Exp(  B) | Lowe Uppe  r r |

|  | |  |  |  | Squar  e |  |  |  |  |  |
| --- | --- | --- | --- | --- | --- | --- | --- | --- | --- | --- |
| (Inter cept) | - 14,73  9 | 1,532  7 | - 17,74  3 | - 11,73  5 | 92,47  6 | 1 | ,000 | 3,972  E-7 | 1,970  E-8 | 8,010  E-6 |
| Tele monit  oring | -,379 | ,7724 | - 1,893 | 1,135 | ,241 | 1 | ,623 | ,684 | ,151 | 3,110 |
| Age | ,056 | ,0198 | ,017 | ,095 | 7,916 | 1 | ,005 | 1,057 | 1,017 | 1,099 |
| Gend  er | ,906 | ,3520 | ,216 | 1,596 | 6,629 | 1 | ,010 | 2,475 | 1,242 | 4,933 |
| DBC | 1,447 | ,6063 | ,259 | 2,636 | 5,698 | 1 | ,017 | 4,252 | 1,296 | 13,95  5 |
| Smok ing_st  atus | ,698 | ,3286 | ,054 | 1,342 | 4,508 | 1 | ,034 | 2,009 | 1,055 | 3,826 |
| CCI | ,056 | ,0881 | -,117 | ,228 | ,400 | 1 | ,527 | 1,057 | ,890 | 1,257 |
| (Scal  e) | 1a |  |  |  |  |  |  |  |  |  |
| (Nega tive binom  ial) | 1a |  |  |  |  |  |  |  |  |  |

Dependent Variable: Hospitalizations

Model: (Intercept), Telemonitoring, Age, Gender, DBC, Smoking_status, CCI, offset = ln_followup_time

a. Fixed at the displayed value.

Poisson regression - ED visits with confounders

## Parameter Estimates

| 95% Wald  Confidence Interval | | | | | Hypothesis Test | | | 95% Wald  Confidence Interval for Exp(B) | | |
| --- | --- | --- | --- | --- | --- | --- | --- | --- | --- | --- |
| Paramet er | B | Std. Erro  r | Lower | Upper | Wald Chi-  Square | df | Sig. | Exp (B) | Lower | Upper |
| (Intercep t) | -  10,  802 | 1,7  174 | - 14,168 | -7,436 | 39,559 | 1 | ,00  0 | 2,0  36E  -5 | 7,029E  -7 | ,001 |
| Telemoni toring | -  ,10  6 | 1,0  820 | -2,227 | 2,014 | ,010 | 1 | ,92  2 | ,89  9 | ,108 | 7,495 |
| Age | ,00  7 | ,02  83 | -,049 | ,062 | ,058 | 1 | ,80  9 | 1,0  07 | ,953 | 1,064 |
| Gender | ,05  4 | ,61  28 | -1,147 | 1,255 | ,008 | 1 | ,92  9 | 1,0  56 | ,318 | 3,509 |
| DBC | 1,8  00 | 1,0  644 | -,286 | 3,886 | 2,859 | 1 | ,09  1 | 6,0  49 | ,751 | 48,719 |
| Smoking  _status | ,31  5 | ,55  65 | -,776 | 1,406 | ,320 | 1 | ,57  1 | 1,3  70 | ,460 | 4,079 |
| CCI | -  ,48  0 | ,29  73 | -1,063 | ,103 | 2,608 | 1 | ,10  6 | ,61  9 | ,346 | 1,108 |
| (Scale) | 1a |  |  |  |  |  |  |  |  |  |

Dependent Variable: ED_visits

| Model: (Intercept), Telemonitoring, Age, Gender, DBC, Smoking_status, CCI, offset = ln_followup_time |
| --- |
| a. Fixed at the displayed value. |

Negative binomial regression - exacerbations with confounders

## Parameter Estimates

| 95% Wald Confidence  Interval | | | | | Hypothesis Test | | | 95% Wald Confidence  Interval for Exp(B) | | |
| --- | --- | --- | --- | --- | --- | --- | --- | --- | --- | --- |
| Parameter | B | Std. Err  or | Lower | Upper | Wald Chi-  Square | df | Sig  . | Ex p(B  ) | Lower | Upper |
| (Intercept) | -  8,6  66 | ,59  84 | -9,838 | -7,493 | 209,69  8 | 1 | ,00  0 | ,00  0 | 5,336E  -5 | ,001 |
| Telemonito  ring | ,76  3 | ,31  50 | ,146 | 1,380 | 5,866 | 1 | ,01  5 | 2,1  45 | 1,157 | 3,976 |
| Age | -  ,00  2 | ,01  00 | -,022 | ,017 | ,051 | 1 | ,82  2 | ,99  8 | ,978 | 1,018 |
| Gender | ,29  0 | ,25  70 | -,214 | ,793 | 1,270 | 1 | ,26  0 | 1,3  36 | ,807 | 2,211 |
| DBC | ,97  3 | ,36  32 | ,261 | 1,685 | 7,178 | 1 | ,00  7 | 2,6  46 | 1,299 | 5,391 |
| Smoking_s tatus | -  ,10  2 | ,20  58 | -,505 | ,302 | ,245 | 1 | ,62  1 | ,90  3 | ,603 | 1,352 |
| CCI | ,11  0 | ,07  89 | -,044 | ,265 | 1,961 | 1 | ,16  1 | 1,1  17 | ,957 | 1,304 |
| (Scale) | 1a |  |  |  |  |  |  |  |  |  |
| (Negative  binomial) | 1a |  |  |  |  |  |  |  |  |  |

Dependent Variable: Exacerbations

Model: (Intercept), Telemonitoring, Age, Gender, DBC, Smoking_status, CCI, offset = ln_followup_time

a. Fixed at the displayed value.

Negative binomial regression -Total outpatient consultations with confounders

## Parameter Estimates

| 95% Wald Confidence  Interval | | | | | Hypothesis Test | | | 95% Wald Confidence  Interval for Exp(B) | | |
| --- | --- | --- | --- | --- | --- | --- | --- | --- | --- | --- |
| Parameter | B | Std.  Err or | Lower | Upper | Wald  Chi- Square | df | Sig  . | Ex p(B  ) | Lower | Upper |
| (Intercept) | -  5,3  79 | ,20  54 | -5,782 | -4,977 | 686,21  3 | 1 | ,00  0 | ,00  5 | ,003 | ,007 |
| Telemonito  ring | 1,2  57 | ,12  61 | 1,009 | 1,504 | 99,307 | 1 | ,00  0 | 3,5  13 | 2,744 | 4,498 |
| Age | ,00  0 | ,00  35 | -,007 | ,006 | ,015 | 1 | ,90  1 | 1,0  00 | ,993 | 1,007 |
| Gender | ,11  0 | ,09  94 | -,085 | ,305 | 1,221 | 1 | ,26  9 | 1,1  16 | ,918 | 1,356 |
| DBC | ,15  8 | ,13  06 | -,098 | ,414 | 1,463 | 1 | ,22  6 | 1,1  71 | ,907 | 1,513 |
| Smoking_s  tatus | ,06  0 | ,07  45 | -,086 | ,206 | ,658 | 1 | ,41  7 | 1,0  62 | ,918 | 1,229 |
| CCI | -  ,01  2 | ,03  66 | -,084 | ,060 | ,112 | 1 | ,73  8 | ,98  8 | ,919 | 1,061 |
| (Scale) | 1a |  |  |  |  |  |  |  |  |  |
| (Negative  binomial) | 1a |  |  |  |  |  |  |  |  |  |

Dependent Variable: Outpatient_and_telephone

Model: (Intercept), Telemonitoring, Age, Gender, DBC, Smoking_status, CCI, offset = ln_followup_time

a. Fixed at the displayed value.

Negative binomial regression - Telephone and screen-to-screen consultations with confounders

## Parameter Estimates

| 95% Wald Confidence  Interval | | | | | Hypothesis Test | | | 95% Wald Confidence  Interval for Exp(B) | | |
| --- | --- | --- | --- | --- | --- | --- | --- | --- | --- | --- |
| Parameter | B | Std. Err  or | Lower | Upper | Wald Chi-  Square | df | Sig  . | Ex p(B  ) | Lower | Upper |
| (Intercept) | -  6,1  45 | ,25  23 | -6,639 | -5,650 | 592,98  5 | 1 | ,00  0 | ,00  2 | ,001 | ,004 |
| Telemonito  ring | 1,9  68 | ,13  68 | 1,700 | 2,236 | 206,91  6 | 1 | ,00  0 | 7,1  55 | 5,472 | 9,355 |
| Age | -  ,00  6 | ,00  44 | -,015 | ,003 | 1,906 | 1 | ,16  7 | ,99  4 | ,985 | 1,003 |
| Gender | ,19  1 | ,12  31 | -,050 | ,433 | 2,420 | 1 | ,12  0 | 1,2  11 | ,951 | 1,541 |
| DBC | ,01  4 | ,16  06 | -,301 | ,329 | ,007 | 1 | ,93  2 | 1,0  14 | ,740 | 1,389 |
| Smoking_s  tatus | ,10  3 | ,09  22 | -,078 | ,283 | 1,237 | 1 | ,26  6 | 1,1  08 | ,925 | 1,327 |
| CCI | ,04  2 | ,04  49 | -,046 | ,130 | ,877 | 1 | ,34  9 | 1,0  43 | ,955 | 1,139 |
| (Scale) | 1a |  |  |  |  |  |  |  |  |  |
| (Negative  binomial) | 1a |  |  |  |  |  |  |  |  |  |

Dependent Variable: Telephone_consultation

Model: (Intercept), Telemonitoring, Age, Gender, DBC, Smoking_status, CCI, offset = ln_followup_time

a. Fixed at the displayed value.

Negative binomial regression - Outpatient clinic visits with confounders

| **Parameter Estimates** | | | | | | |
| --- | --- | --- | --- | --- | --- | --- |
| Parameter | B | Std.  Err or | 95% Wald  Confidence Interval | Hypothesis Test | Ex p(B  ) | 95% Wald  Confidence Interval for Exp(B) |

|  | |  | Lower | Upper | Wald  Chi- Square | df | Sig  . |  | Lower | Upper |
| --- | --- | --- | --- | --- | --- | --- | --- | --- | --- | --- |
| (Intercept) | -  6,1  41 | ,24  36 | -6,618 | -5,664 | 635,52  7 | 1 | ,00  0 | ,00  2 | ,001 | ,003 |
| Telemonito  ring | ,17  3 | ,15  66 | -,134 | ,480 | 1,226 | 1 | ,26  8 | 1,1  89 | ,875 | 1,616 |
| Age | ,00  6 | ,00  41 | -,002 | ,014 | 2,103 | 1 | ,14  7 | 1,0  06 | ,998 | 1,014 |
| Gender | ,02  9 | ,11  35 | -,194 | ,251 | ,064 | 1 | ,80  0 | 1,0  29 | ,824 | 1,285 |
| DBC | ,17  2 | ,15  03 | -,123 | ,467 | 1,309 | 1 | ,25  3 | 1,1  88 | ,885 | 1,595 |
| Smoking_s  tatus | ,06  7 | ,08  50 | -,100 | ,233 | ,621 | 1 | ,43  1 | 1,0  69 | ,905 | 1,263 |
| CCI | -  ,05  5 | ,04  14 | -,136 | ,026 | 1,762 | 1 | ,18  4 | ,94  7 | ,873 | 1,027 |
| (Scale) | 1a |  |  |  |  |  |  |  |  |  |
| (Negative  binomial) | 1a |  |  |  |  |  |  |  |  |  |

Dependent Variable: Outpatient_clinic_visits

Model: (Intercept), Telemonitoring, Age, Gender, DBC, Smoking_status, CCI, offset = ln_followup_time

a. Fixed at the displayed value.
