# Supplementary material for: Real-World Comparison of Telemonitoring Versus Conventional Care in Patients With Chronic Obstructive Pulmonary Disease and Those With Asthma—Impact on Clinical Outcomes and Patient Characteristics: Retrospective Cohort Study
Source: J Med Internet Res. 2025 Aug 14;27:e66743. doi: 10.2196/66743 (PMC12371767; doi:10.2196/66743)
Supplement: Multimedia Appendix 3 [file jmir-v27-e66743-s003.docx]

**Multimedia Appendix 3. Subanalyses asthma/COPD.**

*Asthma
Table 1: characteristics asthma patients*

| **Characteristic** | **Telemonitoring** | **Conventional care** | ***P*-**  **value** |
| --- | --- | --- | --- |
|  | **n = 74** | **n = 366** |  |
|  |  |  |  |
| **Gender, n (%)**   - *Male* - *Female* | 18 (24,3)  56 (75,7) | 150 (41,0)  216 (59,0) | .01 |
| **Age (years), median (IQR)** | 50 (38,75-58,25) | 54 (37,75-67,00) | .03 |
| **BMI^a^, median (IQR)** | 28,88 (25,43-  32,88) | 27,41 (24,09-  32,21) | .17 |
| **Smoking status, n (%)**   - *Never* - *Former* - *Current* | 41 (55,4)  30 (40,5)  3 (4,1) | 187 (51,1)  114 (31,1)  65 (17,8) | .01 |
| **Home status, n (%)**   - *Living alone* - *Living together* | 6 (8,1)  68 (91,9) | 63 (17,2)  303 (82,8) | .049 |
| **Distance to hospital (km), median (IQR)** | 22,67 (10,29-  36,84) | 21,62 (11,49-  31,44) | .83 |
| **Place of residence, n (%)**   - *Urban* - *Rural* | 40 (54,1)  34 (45,9) | 179 (48,9)  187 (51,1) | .42 |
| **Charlson comorbidity index (points), median (IQR)** | 1,00 (1,00-1,25) | 1,00 (1,00-2,00) | .09 |
| **FEV1 % pre^b^, median (IQR)** | 88,13 (76,75-  99,00) | 86,59 (74,00-  99,00) | .74 |

n: number; IQR: Inter Quartile Range; SD: Standard Deviation

a: Body Mass Index (kg/m2)

b: FEV1 % pre: predicted forced expiratory volume in 1 second (%)

*Table 2: Clinical outcomes asthma patients*

| **Clinical outcome** | **Telemonitoring IR** | **Conventional care IR** | **IRR (95% CI)** | ***P*-value** |
| --- | --- | --- | --- | --- |
|  | **n/personyear** | **n/personyear** |  |  |
|  |  |  |  |  |
| **Hospitalizations^a^** | 0.00 | 0.02 | - | - |
| **Emergency department visits^a^** | 0.00 | 0.01 | - | - |
| **Moderate exacerbations^b^** | 0.15 | 0.08 | 1.92 (0.86-4.31) | .11 |
| **Total outpatient consultations^a^**   - **Outpatient clinic visits^a^** - **Telephone and screen-to- screen consultations^a^** | 5.74 | 1.85 | 3.11 (2.34-4.14) | <.001 |
|  | 1.28 | 1.04 | 1.20 (0.85-1.71) | .30 |
|  | 4.46 | 0.81 | 5.59 (4.10-7.63) | <.001 |

n : number; IR: Incidence Rate; IRR: Incidence Rate Ratio; CI: Confidence Interval

a: Asthma/COPD related

b: Prescribed antibiotics or prednisone for exacerbation not resulting in ED visit or hospitalization

*Table 3: Clinical outcomes asthma patients, adjusted for possible confounders^a^*

| **Clinical outcome** | **Telemonitoring IR** | **Conventional care IR** | **IRR (95% CI)** | ***P*-value** |
| --- | --- | --- | --- | --- |
|  | **n/personyear** | **n/personyear** |  |  |
|  |  |  |  |  |
| **Hospitalizations^b^** | 0.00 | 0.02 | - | - |
| **Emergency department visits^b^** | 0.00 | 0.01 | - | - |
| **Moderate exacerbations^c^** | 0.15 | 0.08 | 1.94 (0.85-4.47) | .12 |
| **Total outpatient consultations^b^**   - **Outpatient clinic visits^b^** - **Telephone and screen-to- screen consultations^b^** | 5.74 | 1.85 | 3.06 (2.29-4.10) | <.001 |
|  | 1.28 | 1.04 | 1.19 (0.83 – 1.71) | .34 |
|  | 4.46 | 0.81 | 5.50 (4.00-7.57) | <.001 |

n : number; IR: Incidence Rate; IRR: Incidence Rate Ratio; CI: Confidence Interval
*^a^: Confounders*: age, gender, comorbidity burden and smoking status

b: Asthma/COPD related

c: Prescribed antibiotics or prednisone for exacerbation not resulting in ED visit or hospitalization

*COPD
Table 4: Characteristics COPD patients*

| **Characteristic** | **Telemonitoring** | **Conventional care** | ***P*-**  **value** |
| --- | --- | --- | --- |
|  | **n = 22** | **n = 248** |  |
|  |  |  |  |
| **Gender, n (%)**   - *Male* - *Female* | 11 (50%)  11 (50%) | 124 (50%)  124 (50%) | 1.00 |
| **Age (years), mean (SD)** | 65,77 (10,48) | 69,65 (9,97) | .11 |
| **BMI^a^, median (IQR)** | 24,90 (20,38-  28,38) | 25,88 (22,00-  28,73) | .45 |
| **Smoking status, n (%)**   - *Never* - *Former* - *Current* | 2 (9,1%)  13 (59,1%)  7 (31,8%) | 3 (1,2%)  114 (46,0%)  131 (52,8%) | .01 |
| **Home status, n (%)**   - *Living alone* - *Living together* | 9 (40,9%)  13 (59,1%) | 89 (35,9%)  159 (64,1%) | .64 |
| **Distance to hospital (km), median (IQR)** | 23,26 (15,06-  28,77) | 21,13 (10,14-  28,77) | .58 |
| **Place of residence, n (%)**   - *Urban* - *Rural* | 10 (45,5%)  12 (54,5%) | 133 (53,6%)  115 (46,4%) | .46 |
| **Charlson comorbidity index (points), median (IQR)** | 2,00 (1,00-4,00) | 2,00 (1,00-4,00) | .55 |
| **COPD GOLD^b^, n (%)**   - *GOLD I* - *GOLD II* - *GOLD III* - *GOLD IV* | 0 (0%)  8 (38,1%)  10 (47,6%)  3 (1,9%) | 9 (3,8%)  103 (43,1%)  106 (44,4%)  21 (8,8%) | .66 |
| **FEV1 % pre^c^, median (IQR)** | 48,45 (39,00-  59,02) | 54,00 (41,68-  65,00) | .14 |

n: number; IQR: Inter Quartile Range; SD: Standard Deviation

a: Body Mass Index (kg/m2)

b: GOLD: Chronic Obstructive Lung Disease staging

c: FEV1 % pre: predicted forced expiratory volume in 1 second (%)

*Table 5: Clinical outcomes COPD patients*

| **Clinical outcome** | **Telemonitoring IR** | **Conventional care IR** | **IRR (95% CI)** | ***P*-value** |
| --- | --- | --- | --- | --- |
|  | **n/personyear** | **n/personyear** |  |  |
|  |  |  |  |  |
| **Hospitalizations^a^** | 0.11 | 0.21 | 0.55 (0.13-2.45) | .44 |
| **Emergency department visits^a^** | 0.06 | 0.04 | 1.45 (0.18-11.63) | .72 |
| **Moderate exacerbations^b^** | 0.45 | 0.20 | 2.46 (1.02-4.94) | .045 |
| **Total outpatient consultations^a^**   - **Outpatient clinic visits^a^** - **Telephone and screen-to- screen consultations^a^** | 9.37 | 2.03 | 4.71 (2.94-7.57) | <.001 |
|  | 1.53 | 1.35 | 1.01 (0.61-1.99) | .76 |
|  | 7.78 | 0.68 | 11.92 (7.25-19.61) | <.001 |

n: number; IR: Incidence Rate; IRR: Incidence Rate Ratio; CI: Confidence Interval

a: Asthma/COPD related

b: Prescribed antibiotics or prednisone for exacerbation not resulting in ED visit or hospitalization

*Table 6: Clinical outcomes COPD patients, adjusted for possible confounders*

| **Clinical outcome** | **Telemonitoring IR** | **Conventional care IR** | **IRR (95% CI)** | ***P*-value** |
| --- | --- | --- | --- | --- |
|  | **n/personyear** | **n/personyear** |  |  |
|  |  |  |  |  |
| **Hospitalizations^a^** | 0.11 | 0.21 | 1.07 (0.23-5.07) | .93 |
| **Emergency department visits^a^** | 0.06 | 0.04 | 1.95 (0.23-16.54) | .54 |
| **Moderate exacerbations^b^** | 0.45 | 0.20 | 2.44 (0.96-6.22) | .06 |
| **Total outpatient consultations^a^**   - **Outpatient clinic visits^a^** - **Telephone and screen-to- screen consultations^a^** | 9.37 | 2.03 | 4.92 (3.00-8.06) | <.001 |
|  | 1.53 | 1.35 | 1.11 (0.61-2.04) | .73 |
|  | 7.78 | 0.68 | 13.44 (7.84- 23.04) | <.001 |

n: number; IR: Incidence Rate; IRR: Incidence Rate Ratio; CI: Confidence Interval

a: Asthma/COPD related

b: Prescribed antibiotics or prednisone for exacerbation not resulting in ED visit or hospitalization
